# Morphometric analysis of airways in pre-COPD and mild COPD lungs using continuous surface representations of the bronchial lumen

**DOI:** 10.3389/fbioe.2023.1271760

**Published:** 2023-12-19

**Authors:** David Ortiz-Puerta, Orlando Diaz, Jaime Retamal, Daniel E. Hurtado

**Affiliations:** ^1^ Department of Structural and Geotechnical Engineering, School of Engineering, Pontificia Universidad Católica de Chile, Santiago, Chile; ^2^ Institute for Biological and Medical Engineering, Schools of Engineering, Medicine and Biological Sciences, Pontificia Universidad Católica de Chile, Santiago, Chile; ^3^ Department of Intensive Care Medicine, Pontificia Universidad Católica de Chile, Santiago, Chile

**Keywords:** airway characterization, proximal airways, airway morphometry, luminal volume, luminal eccentricity

## Abstract

**Introduction:** Chronic Obstructive Pulmonary Disease (COPD) is a prevalent respiratory disease that presents a high rate of underdiagnosis during onset and early stages. Studies have shown that in mild COPD patients, remodeling of the small airways occurs concurrently with morphological changes in the proximal airways. Despite this evidence, the geometrical study of the airway tree from computed tomography (CT) lung images remains underexplored due to poor representations and limited tools to characterize the airway structure.

**Methods:** We perform a comprehensive morphometric study of the proximal airways based on geometrical measures associated with the different airway generations. To this end, we leverage the geometric flexibility of the Snakes IsoGeometric Analysis method to accurately represent and characterize the airway luminal surface and volume informed by CT images of the respiratory tree. Based on this framework, we study the airway geometry of smoking pre-COPD and mild COPD individuals.

**Results:** Our results show a significant difference between groups in airway volume, length, luminal eccentricity, minimum radius, and surface-area-to-volume ratio in the most distal airways.

**Discussion:** Our findings suggest a higher degree of airway narrowing and collapse in COPD patients when compared to pre-COPD patients. We envision that our work has the potential to deliver a comprehensive tool for assessing morphological changes in airway geometry that take place in the early stages of COPD.

## 1 Introduction

Chronic obstructive pulmonary disease (COPD) is the third leading cause of death worldwide, representing a critical public health problem with increasing prevalence (Agustí et al., 2023; GOLD, 2023). COPD genesis has long been related to long-term exposure to inhaled pollutants, with cigarette smoke being the most correlated with the onset and progression of the disease (Ananth and Hurst, 2023). Two essential elements of COPD are emphysematous destruction of the lung parenchyma and chronic airway inflammation. These manifestations are responsible for the loss of pulmonary function, evidenced by reduced expiratory airflow and air trapping (Celli et al., 2022).

Small airway disease represents the first stage of development in COPD, preceding emphysematous changes (McDonough et al., 2011; Koo et al., 2018; Labaki et al., 2019). In explanted lungs from COPD patients, micro-computed tomography and histologic examination of terminal bronchioles in areas with variable emphysema severity showed that the narrowing and loss of these airways occurred before alveolar destruction (McDonough et al., 2011). A significant proportion of terminal and transitional bronchioles were lost in lung samples from patients with COPD without signs of emphysema (McDonough et al., 2011; Koo et al., 2018), while the remaining small airways evidenced remodeling changes (McDonough et al., 2011). Findings from Labaki et al. (2019) support the results of smaller, prior cross-sectional, and short-term longitudinal studies (Galbán et al., 2012; Boes et al., 2015) by demonstrating that baseline functional small airway disease detected on chest computed tomography (CT) images are independently associated with an increase in emphysema 5 years later. In other words, transitioning from normal lungs or lungs with small airway disease to emphysema was the most frequent progression into the whole spectrum of disease from pre-COPD stages (Labaki et al., 2019). Besides, studies have confirmed that lumen narrowing in terminal bronchioles occurs before the alveolar dimensions increase into the emphysematous range (Hogg and Timens, 2009). The observation that bronchiolar destruction precedes emphysema is also consistent with several reports showing that the early appearance of emphysematous lesions predicts a more rapid decline in lung function (Yuan et al., 2009; Hoesein et al., 2011; Vestbo et al., 2011; Nishimura et al., 2012) because it is compatible with the widespread destruction of the terminal and preterminal bronchioles.

From a medical imaging standpoint, the structural alteration of the lung parenchyma has been studied using CT images of the lungs, which allows for the enhanced assessment of COPD subtypes (Bodduluri et al., 2017). The characterization of loss of functional lung tissue from CT images has been widely employed in COPD diagnosis, as a decrease in lung attenuation areas correlates with a reduction in pulmonary function (Bodduluri et al., 2013). In contrast, the radiological assessment of airway remodeling has received far less attention, as image resolution and computational tools to analyze airway morphology still represent a technological challenge (Díaz, 2018). Although small airways with a diameter less than 2 *mm* are too small to be detected in clinical CT images, two particular changes in structural features of large airways have been associated with small airway disease. Firstly, using CT imaging and histological analysis, Nakano et al. (Nakano et al., 2005) showed that the wall area in larger airways correlates to the same measure in small airways with an internal diameter of 1.27 *mm* in non-obstructed and moderately obstructed patients. Secondly, the total airway count (TAC) shows a strong correlation with the number of terminal bronchioles, as observed in micro-CT images in excised lung specimens (Kirby et al., 2020). These findings suggest that the remodeling process that affects the peripheral airways in COPD also affects central airways with diameters greater than 2 *mm*.

The morphological study of central airways in COPD patients has been mainly focused on assessing the lumen area and bronchial wall thickness because these structural parameters are related to airflow limitations (Dudurych et al., 2022). As a result, a close association between reduced lumen area, increased bronchial wall area, and decreased predicted forced expiratory volume after one second (FEV_1_%) has been found (Diaz et al., 2016; Bhatt et al., 2019). However, these measurements are estimated by averaging a small selection of cross-section planes perpendicular to the centerline of the airway segment (Coxson, 2008). Such approximations leave out a more precise characterization of the geometry of the airway lumen in terms of irregularities that make it non-circular (Eskandari et al., 2013; 2016), which have been associated with the reduced FEV_1_% (Choi et al., 2015; Choi et al., 2017). This has motivated the use of measures considering the total volume of the lumen, as it might better describe the airway morphology. Diaz et al. (2015) studied lumen volume in the estimation of the bronchial size and reported an association of reduced airway volume with decreased expiratory airflow in never-smokers. Further, Koyama et al. (2012b), Koyama et al. (2012a) found a correlation between the reduction of the airway volume and the reduction of expiratory airflow in COPD patients with different levels of severity. Other volumetric measures, such as the surface-area to lumen volume ratio (SA:V), have been proposed to characterize airway remodeling in a 5-year longitudinal study (Bodduluri et al., 2021). The estimation of these metrics considered the entire airway tree, which does not offer a localized analysis to detect regions of substantial morphological changes that may locally affect airflow.

The approach to geometrical modeling of the human airways uses advanced computational geometry techniques (Tawhai et al., 2009). In particular, techniques for generating surface representations of the airway lumen based on CT imaging should capture the irregular lumen shapes found in the airway tree of lungs with COPD (Choi et al., 2017; Bodduluri et al., 2018), and asthma (Eskandari et al., 2015; Miyawaki et al., 2017). Recently, our group has developed a computational framework for the creation of geometric models of the airway tree from CT images that offers arbitrary and flexible lumen geometries using non-uniform rational B-splines (NURBS) (Ortiz-Puerta et al., 2022). This approach, which we have termed Snakes Isogeometric Analysis (SIGA), has shown high accuracy in the surface representation of healthy and COPD airway trees, as measured in terms of DICE scores. Due to its geometrical foundation, the SIGA method enables accurate and flexible surface analysis suitable for studying complex airway morphologies.

In this work, our objective is to study the morphology of the airway tree using flexible surface representations to understand morphological changes in the early stages of COPD. To this end, we leverage the SIGA method to geometrically analyze the airways of a group of smoking pre-COPD subjects and a group of mild COPD [Global Initiative for Chronic Obstructive Lung Disease (GOLD) I] patients (Agustí et al., 2023) and assess their differences. In Section 2, we briefly revisit the SIGA method and define morphometric volume, surface, and cross-section measures that capture non-circular features of the lumen. In Section 3, we construct surface representations of the airway trees of each individual in the two study groups, from which we estimate and compare the proposed morphometric measures. We examine our results in Section 4, where we offer a discussion about the main conclusions reached and highlight aspects of this work that can be improved in future contributions.

## 2 Geometrical modeling and morphometric metrics for the analysis of the respiratory airways

### 2.1 Airway surface modeling: the Snakes isogeometric analysis (SIGA) method

**FIGURE 1:**
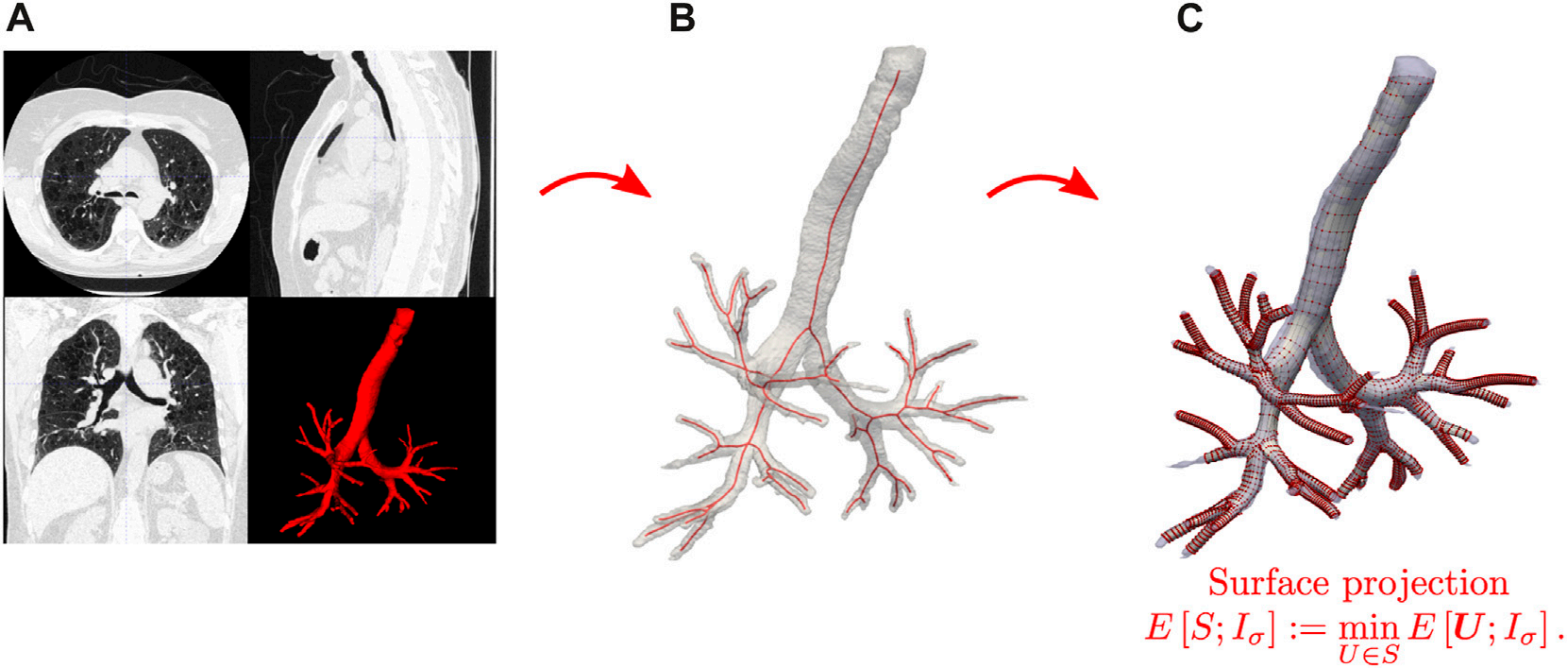
Schematic of the SIGA method for creating geometrical models of the airways. **(A)** CT image processing and segmentation. **(B)** Skeletonization of binary airway images. **(C)** Surface construction and projection from solving the Snakes segmentation problem.

In the following, we briefly describe the SIGA method for constructing geometrical surface models of the respiratory airways (Ortiz-Puerta et al., 2022), see Figure 1 for a schematic. The first step considers volumetric CT images of the lungs in end-expiration (EE) and end-inspiration (EI) stages, from which we extract binary masks using the image level-set-based segmentation tool provided by the ITK-SNAP software (Yushkevich et al., 2006), see Figure 1A. We perform a visual corroboration of the result to correct possible leakage of the level-set into the parenchyma using the ITK-SNAP editing tools. This leakage can happen mainly in smaller airways with poorly defined bronchial walls due to resolution limitations. Furthermore, considering the voxel size of the images (0.7 *mm*), we established not to segment airways with less than three voxels (2.1 *mm*) in diameter to avoid leakage in smaller airways.

From this step, we obtain binary images $I_{\mathrm{seg}}:\Omega \subset R^{3}\to \left\{0,1\right\}$ in NIFTI format with the same voxel size and dimensions as the CT images, where the luminal volume of the airways is marked by ones and the rest by zeroes. Then, using the CGAL library (Gao et al., 2020), we calculate the skeletons from the binary image, see Figure 1B. We identify each airway segment delimited by the bifurcation nodes in the skeleton and recursively identify their child branches to mark them by generation for further spatial grouping. We further implement the approach set forth by Miyawaki et al. (Miyawaki et al., 2017) to post-process the skeleton to identify short branches and create fork-type trifurcations. This methodology involves identifying the child branches from a parent and comparing the proportion between their length and radius, i.e., *p*_*c*_ = *l*_*c*_/*r*_*c*_, where *l*_*c*_ is the child branch length and *r*_*c*_ is the radius. If one of them is too short with respect to their radius, i.e., *p*_*c*_ < 0.3, we transform the bifurcation into a trifurcation by identifying the short branch children and connecting them to the short branch parent through the bifurcation nodes. We refer the reader to Miyawaki et al. (2017) for a detailed description of this post-processing step.

In the next step, the skeleton nodes of each airway branch are used to align octagonal templates containing the spatial control points that will represent cylindrical surfaces. The resulting control points are connected to create the control point mesh necessary to represent the NURBS cylindrical surfaces. These surfaces are then evolved to model the airway boundary by solving variational formulation of the Snakes problem Kass et al. (1988), see Figure 1C. Let $\hat{\Omega }\subset R^{2}$ and $S≔{\left[H^{2}\left(\hat{\Omega },R\right)\right]}^{3}$ be the space of surface embeddings on the physical domain $R^{3}$ . Let $I_{\mathrm{seg}}:\Omega \subset R^{3}\to \left\{0,1\right\}$ be a volumetric image; The Snakes problem can be stated as follows: Find the optimal surface S∈S such that

$$\begin{array}{cc} E\left[S;I_{\sigma }\right]≔\underset{U\in S}{\min}\left[\frac{\alpha }{2}\int_{\hat{\Omega }}\left(\|U_{,\xi }{\|}^{2}+\|U_{,\eta }{\|}^{2}\right)d\hat{\Omega }\right.\\ & +\frac{\beta }{2}\int_{\hat{\Omega }}(\|U_{,\xi \xi }{\|}^{2}+\|U_{,\xi \eta }{\|}^{2}+\|U_{,\eta \eta }{\|}^{2})d\hat{\Omega }-\frac{\lambda }{2}\int_{\hat{\Omega }}\|\nabla \left(G_{\sigma }*I_{\mathrm{seg}}\right)\left(U\right){\|}^{2}d\hat{\Omega }], \end{array}$$ (1)

where *α*, *β* > 0 are parameters that control the regularizing terms, *λ* > 0 is the image energy parameter, $U_{,\mu }≔\frac{\partial U}{\partial \mu }$ and $U_{,\mu \nu }≔\frac{\partial^{2}U}{\partial \mu \partial \nu }$ are the first and second derivatives respect the parametric coordinates *μ*, *ν* ∈ {*ξ*, *η*}, and *G*_σ_*I_*seg*_ is a Gaussian filter applyed to the binary image *I*_*seg*_. Note that the Gaussian filter is used to smooth the boundaries of the binary image and not to reduce noise, and in the following, we referred to the filtered image as *I*_*σ*_. Then, we define the space $S_{t}≔{\left[H^{2}\left(\hat{\Omega },R\right)\right]}^{3}\times R^{+}$ , where *t* is a fictitious time variable, and rate potential $\Psi \left[\dot{U}\right]≔\frac{1}{2}\int_{\hat{\Omega }}|\dot{U}{|}^{2}d\hat{\Omega }$ . Thus, the evolution of the surface can be expressed by the following variational gradient-flow formulation (Hurtado and Henao, 2014): Find $S\in S_{t}$ such that

$$D_{\nu }\Psi \left[\dot{S}\right]+D_{\nu }E\left[S;I_{\sigma }\right]=0,\;\forall \nu \in S.$$ (2)

where $D_{\nu }E\left[S\right]≔\frac{\partial }{\partial \epsilon }E\left[S+\epsilon \nu \right]{|}_{\epsilon =0}$ is the Gateaux differential of an arbitrary functional *E*. For the spatial discretization of Eq. 2, we use a multi-patch approach. To this end, we define the NURBS trial and test subspaces in Eq. 3, which read

$$\begin{array}{cc} S_{t}^{h} & :=\left\{S^{h}\left(\cdot ,t\right)\in {\left[H^{2}\left({\hat{\Omega }}_{\rho },R\right)\right]}^{3}\times R^{+}:S^{h}\left(\cdot ,t\right){|}_{{\hat{\Omega }}_{\rho }}\in Q\left({\hat{\Omega }}_{\rho }\right),\forall \rho =1,\ldots ,n_{pt}\right\},\\ V^{h} & :=\left\{\nu^{h}\in {\left[H^{2}\left({\hat{\Omega }}_{\rho },R\right)\right]}^{3}:\nu^{h}{|}_{{\hat{\Omega }}_{\rho }}\in Q\left({\hat{\Omega }}_{\rho }\right),\forall \rho =1,\ldots ,n_{pt}\right\}, \end{array}$$ (3)

where *n*_*pt*_ is the number of patches, $S_{t}^{h}\subset S_{t}$ , $V^{h}\subset S$ , and $Q\left({\hat{\Omega }}_{\rho }\right)$ is the space spanned by isogeometric shape functions that result from the 2-tensor product of 1D NURBS functions. Then, the multi-patch NURBS surface discretization $S^{h}{\left|\right.}_{{\hat{\Omega }}_{\rho }}≔S_{\rho }^{h}$ and $\nu^{h}{\left|\right.}_{{\hat{\Omega }}_{\rho }}≔\nu_{\rho }^{h}$ with normalized patch domain ${\hat{\Omega }}_{\rho }≔{\left[0,1\right]}^{2}$ , takes the form

$$S_{\rho }^{h}=\overset{n_{\rho \eta }}{\underset{j=1}{\sum }}\overset{n_{\rho \xi }}{\underset{i=1}{\sum }}R_{i,j}^{p,q}\left(\xi_{\rho },\eta_{\rho }\right)b_{i,j}^{\rho }\left(t\right),$$ (4)

$$\nu_{\rho }^{h}=\overset{n_{\rho \eta }}{\underset{j=1}{\sum }}\overset{n_{\rho \xi }}{\underset{i=1}{\sum }}R_{i,j}^{p,q}\left(\xi_{\rho },\eta_{\rho }\right)d_{i,j}^{\rho },$$ (5)

where $\left(\xi_{\rho },\eta_{\rho }\right)\in {\hat{\Omega }}_{\rho }$ , $b_{i,j}^{\rho }:\left(0,T\right]\to R^{3}$ are the spatial control point defined for the patch *ρ*, and $d_{i,j}^{\rho }\in R^{3}$ . The NURBS basis functions are defined in Eq. 6, which take the form

$$R_{i,j}^{p,q}\left(\xi_{\rho },\eta_{\rho }\right)=\frac{N_{i,p}\left(\xi_{\rho }\right)M_{j,q}\left(\eta_{\rho }\right)w_{i,j}^{\rho }}{\sum_{\hat{j}=1}^{n_{\rho \eta }}\sum_{\hat{i}=1}^{n_{\rho \xi }}N_{\hat{i},p}\left(\xi_{\rho }\right)M_{\hat{j},q}\left(\eta_{\rho }\right)w_{\hat{i},\hat{j}}^{\rho }},$$ (6)

where *N*_*i*,*p*_ (*ξ*_*ρ*_), *M*_*j*,*q*_ (*η*_*ρ*_) formed by the B-splines basis with polynomial order *p*, *q*, calculated with the Cox the Boor recursion formula (Hughes et al., 2005), $w_{i,j}\in R^{+}$ the NURBS weights, and *n*_*ρξ*_, *n*_*ρη*_ the number of basis function on each parameter direction. Note that each $S_{\rho }^{h}$ in Eq. 4 defines a surface patch *ρ* in the physical domain and that the spatial control points $b_{i,j}^{\rho }$ are defined for each patch and evolve on time. Using Eqs 4, 5, we get the spatial discretization of problem (Eq. 2). The result is a semi-discrete system of equations that depends continuously on time. Thus, temporal discretization was carried out using a semi-implicit time integration scheme leading to a linear matrix system. Each time step solution of the linear system is the new spatial position of the control points $b_{i,j}^{\rho }$ of the expression (Eq. 4), defining the NURBS surface representation $S_{\rho }^{h}$ of the airways. See Ortiz-Puerta et al. (2022) for further details of the linear system and temporal integration scheme.

To evaluate the performance of the SIGA method and validate the accuracy of the surface representation, we calculate the DICE coefficient of similarity. To this end, we use the binary image *I*_*seg*_ from the initial segmentation step and create the surface-based binary image *I*_*siga*_ where voxels with value 1 are inside of the airways NURBS surfaces. The DICE coefficient is defined in Eq. 7, which reads

$$\text{DICE}=2\frac{\left|I_{\mathrm{seg}}\cap I_{\mathrm{siga}}\right|}{\left|I_{\mathrm{seg}}|+|I_{\mathrm{siga}}\right|},\;\text{DICE}\in \left[0,1\right],$$ (7)

where |⋅| is the volume given by the number of voxels with a value equal to 1 of the binary images. The numerator defines the intersection, i.e., the common voxels between both images, and the denominator is the addition of both volumes. This coefficient measures similarity between the images, where DICE = 1 indicates perfectly matching volumes and DICE = 0 indicates no match. Considering that the voxels with value 1 in binary image *I*_*siga*_ are defined inside the NURBS surface of each branch, the DICE coefficient also validates the accuracy of the geometrical measures as it is defined directly. Previous studies suggest that DICE ≥0.7 can be considered an acceptable threshold to establish similarity (Arrieta et al., 2017).

### 2.2 Morphometric measures for airway analysis

For the morphometry study, we considered the airways as tubular structures with arbitrary cross-section shapes. Each airway segment is identified using the bifurcation nodes of the skeleton and its corresponding NURBS surfaces as in Eq. 4. Based on these representations, we compute the following parameters:

#### 2.2.1 Total and generation airway count

The total airway count (TAC) is the sum of all segments included in the airway-tree representation for a subject. It measures all visually connected airways from which we can extract morphological information (Kirby et al., 2018). The generation airway count (GAC) is the number of airway segments in a particular generation in the lung of one subject. We note that the sum of all GACs equals the TAC.

#### 2.2.2 Segment luminal volume

**FIGURE 2:**
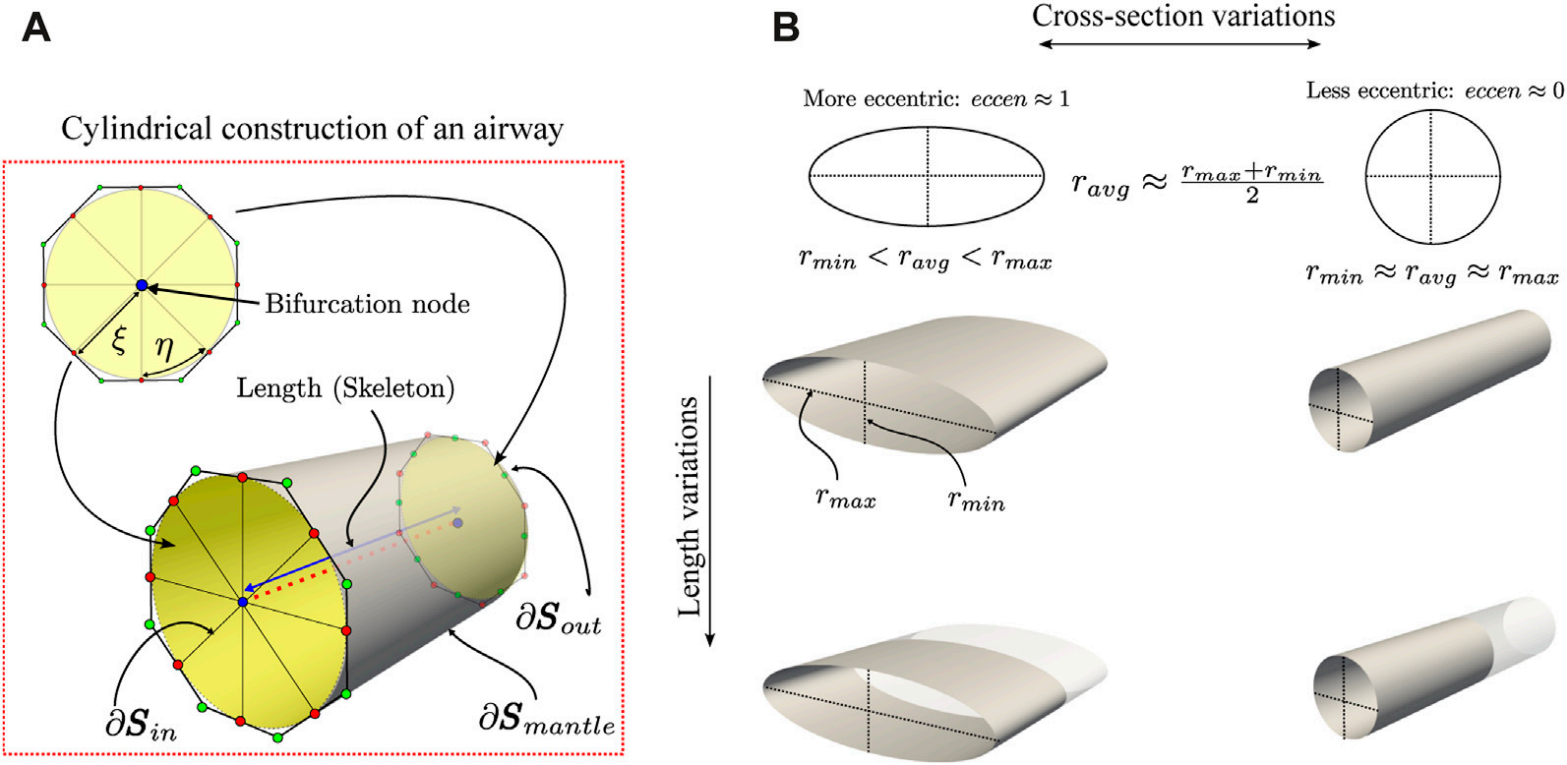
Schematic of the geometrical characterization of the airway morphology. In the ideal case of perfectly cylindrical airways **(A)**, its cross-section has an *eccen* = 0 since the minimum and maximum radius are the same, and its length is given by the skeleton. Also, geometrical properties, such as the volume and surface area to volume ratio (SA:V), are easily calculated using NURBS surfaces. However, the airways are far from ideal and exhibit variations in their cross-section and length **(B)**.

Airways luminal volume cannot be directly calculated since the SIGA method only delivers their surface representation. To calculate the volume, we use the parametric model of NURBS surfaces in Eq. 4 and apply the divergence theorem. However, we must close the surface containing the luminal volume to be completely continuous. To this end, we consider the octagonal templates located at each end of the airway and connect them with their corresponding bifurcation node; see Figure 2A. Then, we create the *inlet* *∂* ***S***_*in*_ and *outlet* *∂* ***S***_*out*_ surfaces using the expression in Eq. 4 and the control points defined by the octagonal mesh, and rename the airway lumen surface as *∂* ***S***_*mantle*_. We remark that to guarantee a minimum *C* ^0^ continuity of the total surface, it is important that for the new surfaces, the *η* parametric direction satisfies the same regularity as the *∂* ***S***_*mantle*_ surface. Now, let $\Omega \subset R^{3}$ be the airway lumen volume. Its surface boundary is such that *∂*Ω = *∂* ***S***_*in*_ ∪ *∂* ***S***_*out*_ ∪ *∂* ***S***_*mantle*_, and clearly $\partial \Omega \subset R^{3}$ . Then, we define the position vector field $X:\Omega \to R^{3}$ where [*x*,*y*,*z*]^*T*^ = ***X*** (Ω) are the position vectors. Taking the divergence of the position vector field ***X*** we have

$$X=\left[\begin{array}{c} x\\ y\\ z \end{array}\right]\Rightarrow \frac{1}{3}divX=1.$$ (8)

Now, we consider the integral expression to calculate the lumen volume of Ω. Then, replacing Eq. 8 and using the Divergence theorem, the lumen volume is expressed in Eq. 9, which takes the form

$$volume=\int_{\Omega }1d\Omega =\frac{1}{3}\int_{\Omega }divXd\Omega =\frac{1}{3}\int_{\partial \Omega }X\cdot nd\partial \Omega ,$$ (9)

where ***n*** outward pointing unit normal vector defined over *∂*Ω. Recalling the union of surfaces boundary *∂*Ω = *∂* ***S***_*in*_ ∪ *∂* ***S***_*out*_ ∪ *∂* ***S***_*mantle*_ and that each surface is defined parametrically as a NURBS surface patch $S_{\rho }^{h}$ , where ρ∈P≔{in,out,mantle} , we have

$$\begin{array}{cc} volume & =\frac{1}{3}\int_{\partial \Omega }X\cdot nd\partial \Omega \\ & =\frac{1}{3}\underset{\rho \in P}{\sum }\left[\int_{\partial S_{\rho }}X_{\rho }\cdot n_{\rho }d\partial S_{\rho }\right],\;\text{with}\;n_{\rho }=\frac{S_{\rho ,\xi }^{h}\times S_{\rho ,\eta }^{h}}{\left\|S_{\rho ,\xi }^{h}\times S_{\rho ,\eta }^{h}\right\|}, \end{array}$$ (10)

where $S_{\rho ,\alpha }^{h}≔\frac{\partial S_{\rho }^{h}}{\partial \alpha }$ , is the partial derivative of the surface with respect each parametric coordinate *α* ∈ {*ξ*, *η*}. Following this, we consider that the dot product ***X***_*ρ*_ ⋅ ***n***_*ρ*_ is defined over the embedded parametrical surfaces *∂* ***S***_*ρ*_. Then, we use the results from Dedè and Quarteroni (2015) and define the Jacobian determinant ${\hat{g}}_{\rho }\left(\xi_{\rho }\right)≔\sqrt{det\left(F{\left(\xi_{\rho }\right)}^{T}F\left(\xi_{\rho }\right)\right)}$ , where $F\left(\xi_{\rho }\right)≔\frac{\partial X_{\rho }}{\partial \xi_{\rho }}$ , and the vectors ***X***_*ρ*_ = (*x*_*ρ*_, *y*_*ρ*_, *z*_*ρ*_) and $\xi_{\rho }=\left(\xi_{\rho },\eta_{\rho }\right)\in {\hat{\Omega }}_{\rho }$ are the spatial point defined by the NURBS surfaces and the parametric coordinates, respectively. Thus, from Eq. 10, we have

$$volume=\frac{1}{3}\underset{\rho \in P}{\sum }\left[\int_{{\hat{\Omega }}_{\rho }}X_{\rho }\cdot n_{\rho }\hat{g}d{\hat{\Omega }}_{\rho }\right],\;P≔\left\{in,out,mantle\right\}.$$ (11)

In this work, volume results are presented in *mm* ^3^.

#### 2.2.3 Surface area to volume ratio (SA:V)

This measure was proposed by Bodduluri et al. (2021) to characterize airway remodeling. It extends the cross-sectional relation between the lumen area and the inner perimeter along the airway extension. We remark that SA:V ratio should increase generation-by-generation until reaching functional tissue as a large alveolar surface area allows for more effective gas exchange (Bodduluri et al., 2021). To estimate this parameter, we calculate the area of the surface boundary *∂*Ω as

$$area=\frac{1}{3}\underset{\rho \in P}{\sum }\left[\int_{{\hat{\Omega }}_{\rho }}|S_{\rho ,\xi }^{h}\times S_{\rho ,\eta }^{h}|d{\hat{\Omega }}_{\rho }\right],\;P≔\left\{in,out,mantle\right\},$$ (12)

with the area expressed in *mm* ^2^. Volume and area integrals (Eqs 11, 12), respectively, are numerically solved using standard Gauss-Legendre quadrature, as detailed in Ortiz-Puerta et al. (2022). Thus, the surface area to volume ratio is then given by Eq. 13, which reads

$$SA:V=\frac{area}{volume},$$ (13)

were *area* and *volume* are given by Eqs 11, 12, respectively, and results are presented in *mm* ^−1^.

#### 2.2.4 Luminal eccentricity

This adimensional measure quantifies the distortion of the cross-section of the airway in terms of the minor and major radii (Oakes et al., 2012). The major and minor radii are calculated from the orthogonal projection of points from the NURBS surfaces to the line segments defined from the internal nodes of the skeleton of each branch. The points on the surface are generated from the parametric coordinates (*ξ*, *η*) in the normalized domain $\hat{\Omega }$ of the mantle surface. Then, for each line segment of the skeleton, we use the orthogonal projection vectors to calculate the minimum, maximum and average distance using the Euclidean norm. Note that in a circular cross-section, these projection vectors have the same size; see Figure 2B. Finally, we calculate the minimum *r*_min_, maximum *r*_max_, and average *r*_*avg*_ radii of the total airway segment by averaging these measures from each skeleton line segment. The luminal eccentricity is then defined in Eq. 14

$$eccen=\sqrt{1-{\left(\frac{r_{\min}}{r_{\max}}\right)}^{2}},\;eccen\in \left[0,1\right).$$ (14)

For a circle, we have *eccen* = 0; for an ellipse, the luminal eccentricity will be between 0 and 1, i.e., *eccen* ∈ (0, 1).

#### 2.2.5 Airway segment length

It corresponds to the Euclidean distance between two connected skeleton nodes of a segment $n_{i}\in {SN}_{b}$ that belong to an airway branch *b*, which is defined in Eq. 15

$$length=\underset{n_{i}\in {SN}_{b}}{\sum }\sqrt{n_{i}^{2}-n_{i+1}^{2}}.$$ (15)

Results are reported in *mm*.

The SIGA method specifically focuses on representing the luminal surface of the airways. Consequently, it is not suitable for measuring bronchial wall thickness. Geometrical measurements are calculated for all available airway segments for inspiratory and expiratory breathing states. In addition, all segments are grouped by generations from generation 0 (trachea) to 5 (subsegmental bronchi). All algorithms and measures presented in this study were implemented in an in-house code using Python 3.9 and on an Intel(R) Core(TM) i7-3770 CPU at 3.40 GHz with 16 GB of RAM workstation.

### 2.3 Statistical analysis

Statistical analysis was performed using SciPy 1.7.3 (Python 3.9). We used the Shapiro-Wilk test to confirm the non-normal distribution of the data. Then, we used the Mann-Whitney U test for the comparative study to find significant differences between both groups for generations 0 to 5, at EE and EI, and for each measure. While it is important to acknowledge that the Mann-Whitney test can lead to type II errors, we find it the most suitable choice for statistical analysis given the sample size and non-normal data distribution.

## 3 Morphometric study of the airways of pre-COPD smoking subjects and mild COPD patients

### 3.1 Experimental groups and image datasets

We analyzed the pulmonary function and image datasets from participants recruited in a study previously reported in the literature (Labarca et al., 2017). We constructed two groups comprising six participants through random selection from a database, with an equal distribution of three males and three females in each group and an age range of 65 ± 6 years. The Control (pre-COPD) group consisted of smokers with no signs of COPD. The COPD group comprised patients diagnosed with COPD at GOLD stages I of severity. We did not conduct a gender-based study, considering the small sample size according to this criterion. Anthropometric and pulmonary function data for these groups are shown in Table 1. Emphysema was measured as the percent of low attenuation areas less than −960 Hounsfield units (LAA%) (Madani et al., 2007).

**TABLE 1:** Anthropometric and pulmonary function data for the groups under study. Data are presented as median ± IQR. *p < 0.05 Mann-Whitney test. mMRC-Dyspnea = modified medical research council, FEV_1_pred = predicted forced expiratory volume in one second, TLCpred = predicted total lung capacity, FRCpred = predicted functional residual capacity, DLCOpred = predicted diffusing capacity for carbon monoxide, LAA = low attenuation area, SMWDpred = predicted 6 min walk distance, FEV_1_/FVC = rate between forced expiratory volume in one second and forced vital capacity.

Parameters	Control	COPD	*p*-value
Participants, n	6	6	1.00
Female sex, %	50	50	1.00
Age; year	62.0 ± 6.0	64.5 ± 4.5	0.57
Height, cm	159.0 ± 4.5	161.5 ± 8.5	0.47
Weight, kg	62.6 ± 6.3	74.5 ± 15.3	0.15
BMI, kg/cm^2^	25.9 ± 4.0	28.7 ± 5.4	0.24
mMRC-Dyspnea	0	1 and 2	0.002*
TLCpred, %	97.1 ± 17.5	109.4 ± 16.5	0.18
FRCpred, %	87.9 ± 12.8	107.2 ± 20.1	0.18
FEV_1_pred, %	118.3 ± 27.9	91.3 ± 22.4	0.025*
DLCOpred, %	88.8 ± 6.8	68.1 ± 17.0	0.008*
LAA, %	0.4 ± 1.1	3.3 ± 12.2	0.013*
SMWDpred, %	95.5 ± 14.3	88.2 ± 8.9	0.041*
FEV_1_/FVC %	79.8 ± 4.5	63.0 ± 12.2	0.002*

**FIGURE 3:**
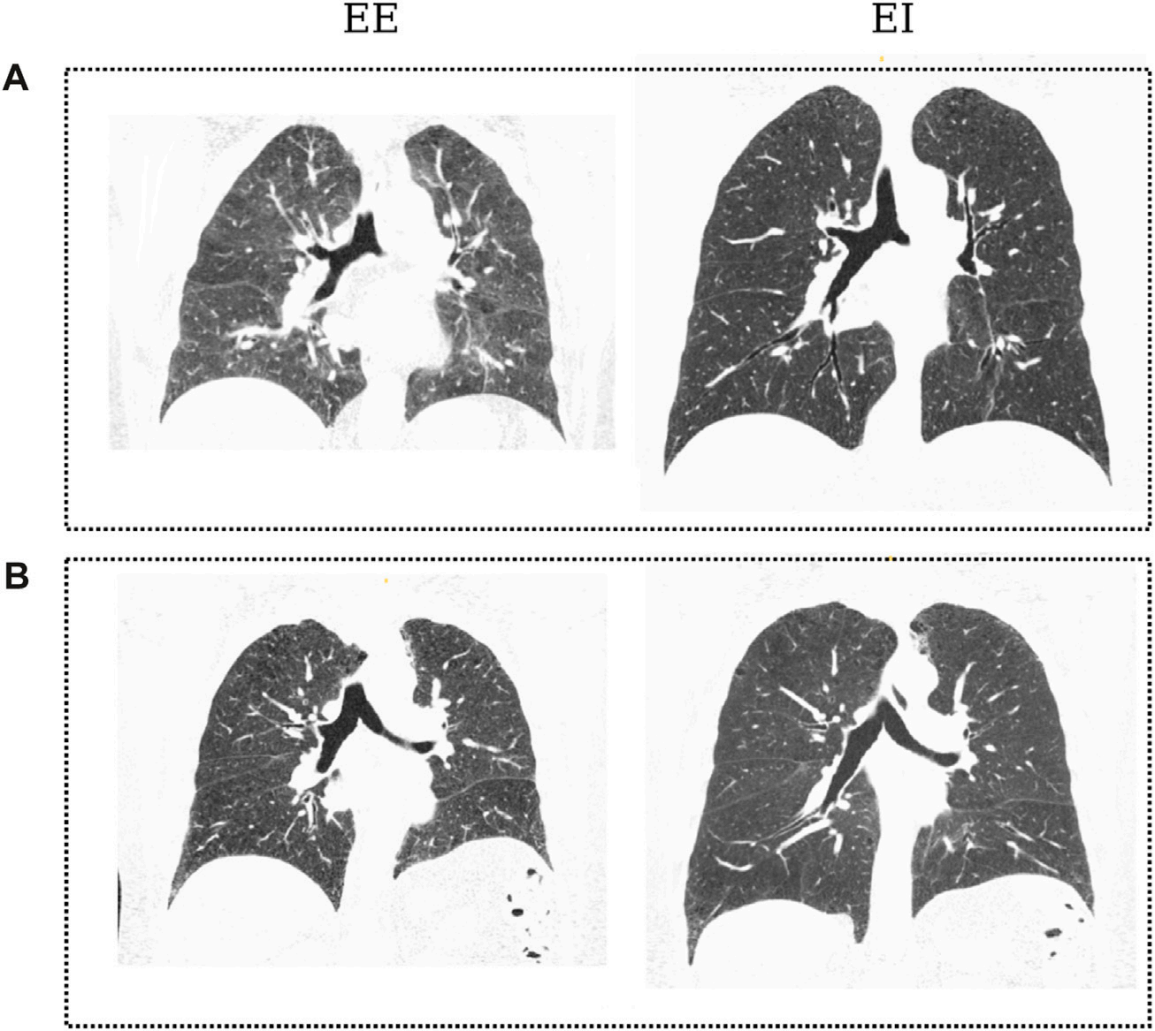
Coronal slices of CT images from patients of both groups in the dataset: Control subject **(A)** at EE and EI; COPD patient **(B)** at End of Expiration (EE) and End of Inspiration (EI). Black regions denote aerated areas in the lungs and the airways.

CT images of the thorax at EE and EI for each participant were retrieved and analyzed in this work. The voxel resolution was 0.7*mm* × 0.7*mm* × 0.5 *mm*. Figure 3 shows coronal planes of representative subjects in each group during EE and EI.

**FIGURE 4:**
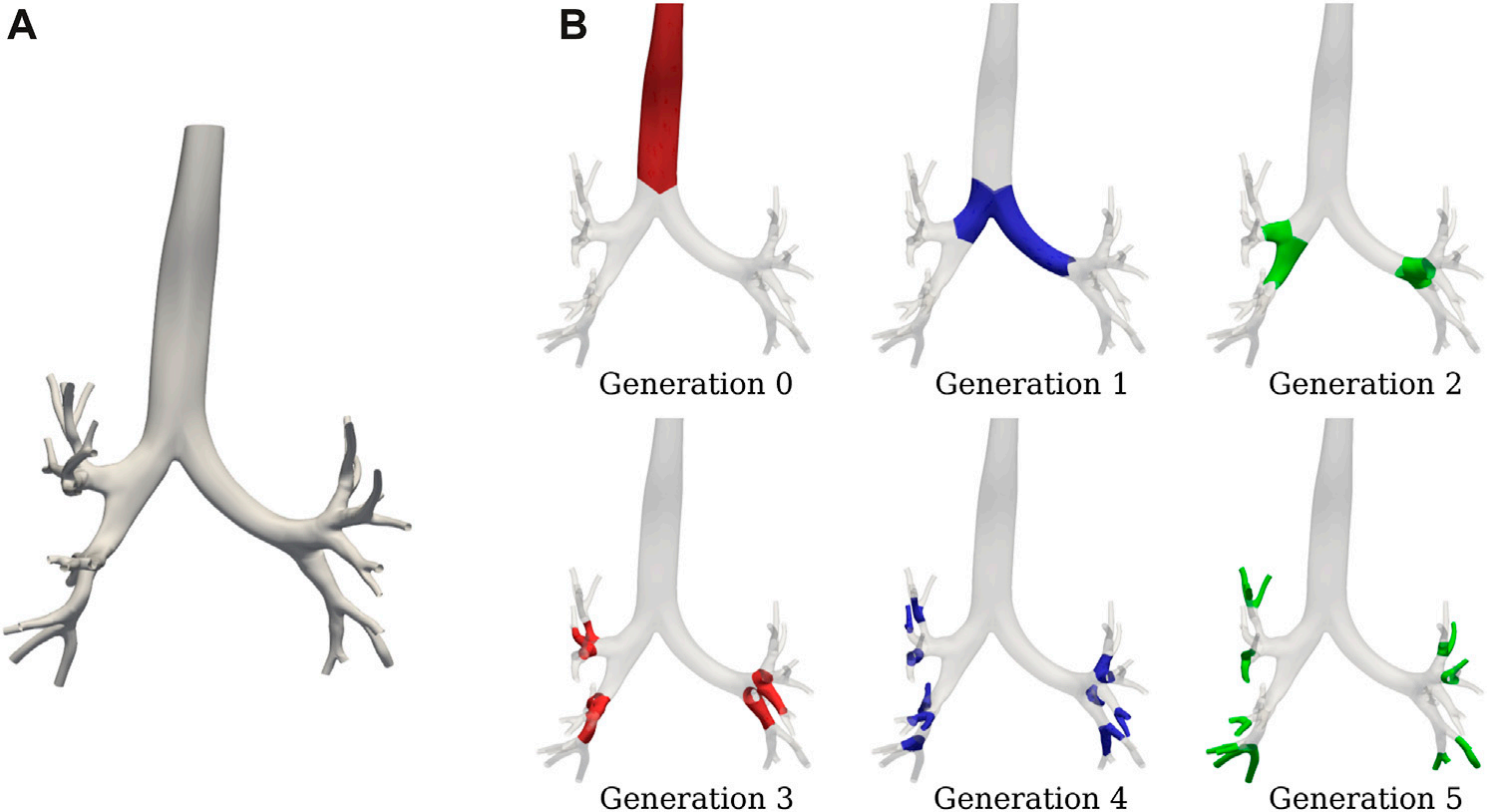
SIGA geometrical model for a COPD patient: **(A)** surface results at EE with DICE coefficient of 0.89, and **(B)** airway spatial grouping from generation 0 to 5.

**FIGURE 5:**
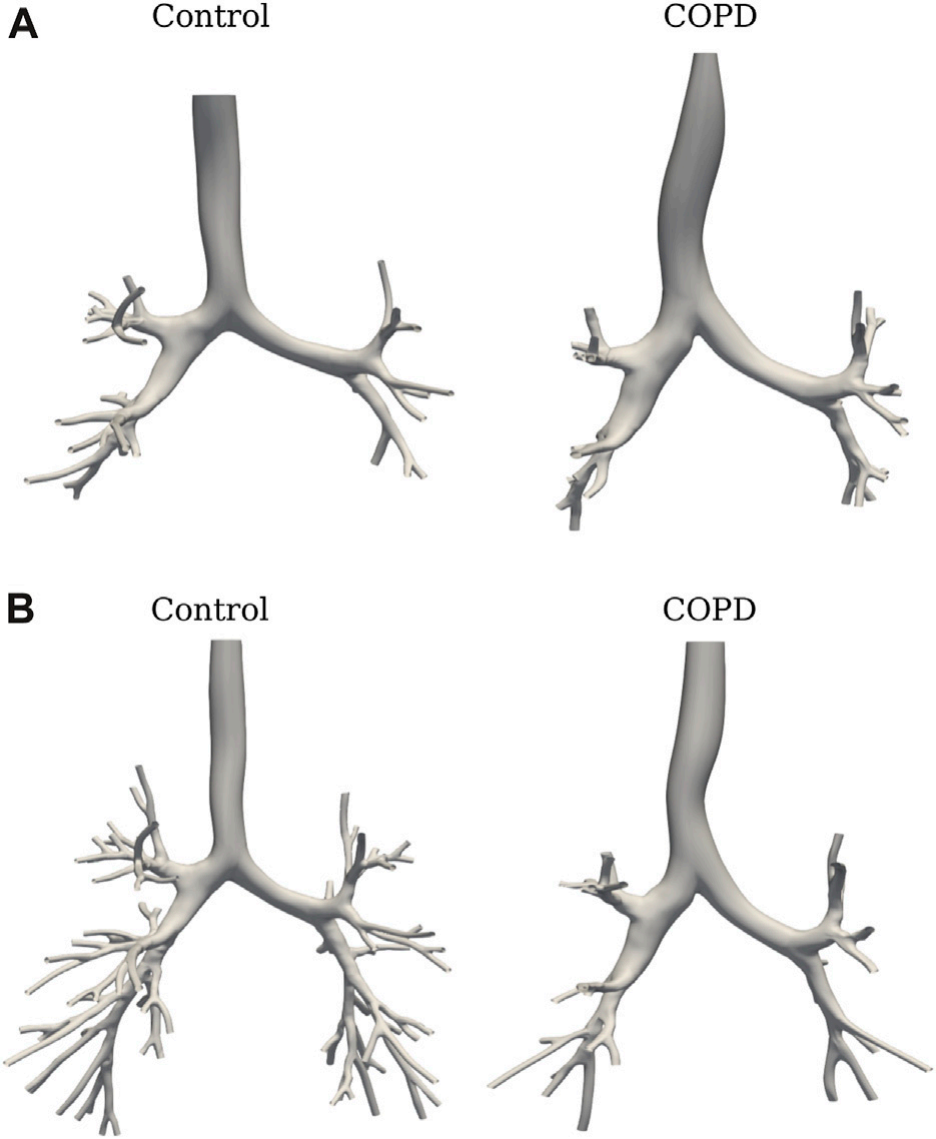
SIGA surface models comparison from subjects of both groups: at EE **(A)**, and EI **(B)**.

To create the geometric airway models, we adjusted the parameters of the SIGA model so that the best surface representation was achieved. To this end, we considered two surface patches (*ρ* = 2) for each airway segment to reduce the lack of smooth regularity in the interface between two adjacent patches (*G* ^1^-continuity) without modifying the control point templates. The accuracy of the representation was measured using the DICE coefficient. We set the polynomial degrees to *p*, *q* = 3 to guarantee a higher regularity (*C* ^2^) inside each patch. Parameters for the energy functional (Eq. 1) were calibrated using the iterative scheme proposed in Ortiz-Puerta et al. (2022), where we sampled the parameter space and picked the point that maximized the DICE coefficient. For the regularity term we used *α* = 10^–2^, *β* = 10^–3^, for the image energy term, *λ* = 0.1, and for the Gaussian filter, *σ* = 2. Time steps were kept to Δ*t* = 0.01 and a maximum of 35 iterations. These parameters yielded an excellent performance, with DICE >0.88 in all the images for both states and groups. The total time for the workflow presented in Figure 1 is 4.5 h, with steps (a) and (c) being the most time-consuming with about 1 and 3.5 h, respectively. Figure 4 shows the SIGA surface results for a COPD patient at EE with the spatial grouping of the airways by generations. Figure 5 presents the results for two patients in both breathing states.

### 3.2 Results

**FIGURE 6:**
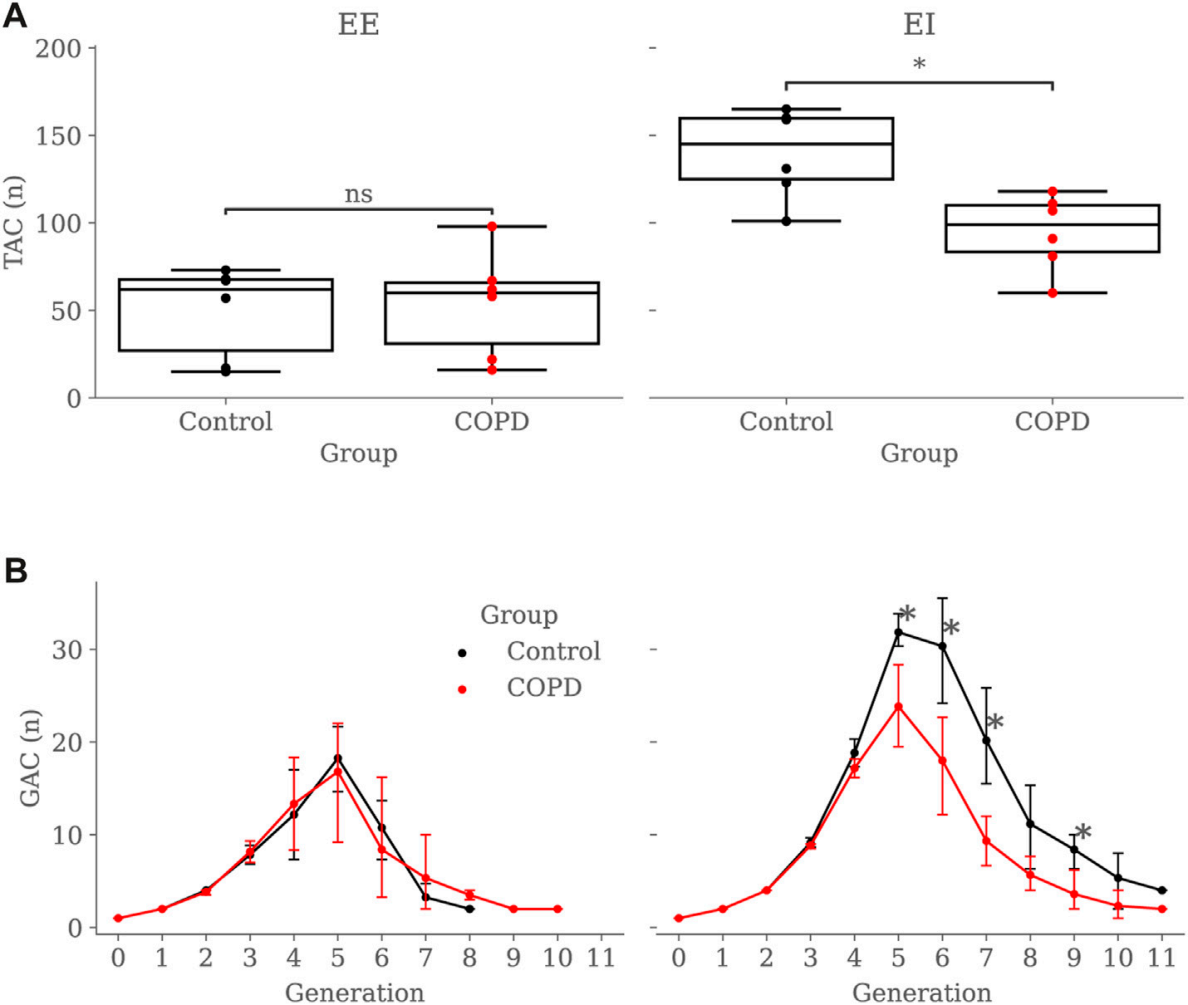
Group comparison of airway counts: **(A)** Total Airway Count (TAC) by group and breathing state, **(B)** Generation Airway Count (GAC) by group and breathing state. In subfigure **(B)**, error bars represent confidence intervals. Nomenclature: ns = no significant differences, *p < 0.05.

Figure 6A shows the TAC comparison. While TAC was similar between groups at EE (49.5 ± 26.4 vs. 53.8 ± 30.5 in Control and COPD groups, respectively), it was significantly higher in the Control group at EI, with values of 139.8 ± 25.6 (Control) and 94.6 ± 21.7 (COPD) (*p* = 0.015). Figure 6B reports a similar comparison for GAC. At EE, GAC was similar between groups. At EI, GAC in the Control group was significantly different from the COPD group for the 5^*th*^ (31.8 ± 2.2 vs. 23.8 ± 5.6, *p* = 0.03), 6^*th*^ (30.3 ± 7.3 vs. 18.0 ± 6.7, *p* = 0.03), 7^*th*^ (20.2 ± 6.4 vs. 9.3 ± 3.3, *p* = 0.008), and 9^*th*^ (8.4 ± 2.3 vs. 3.6 ± 2.7, *p* = 0.03) generations, respectively. See Figure 5 for the airway model of two representative subjects.

**FIGURE 7:**
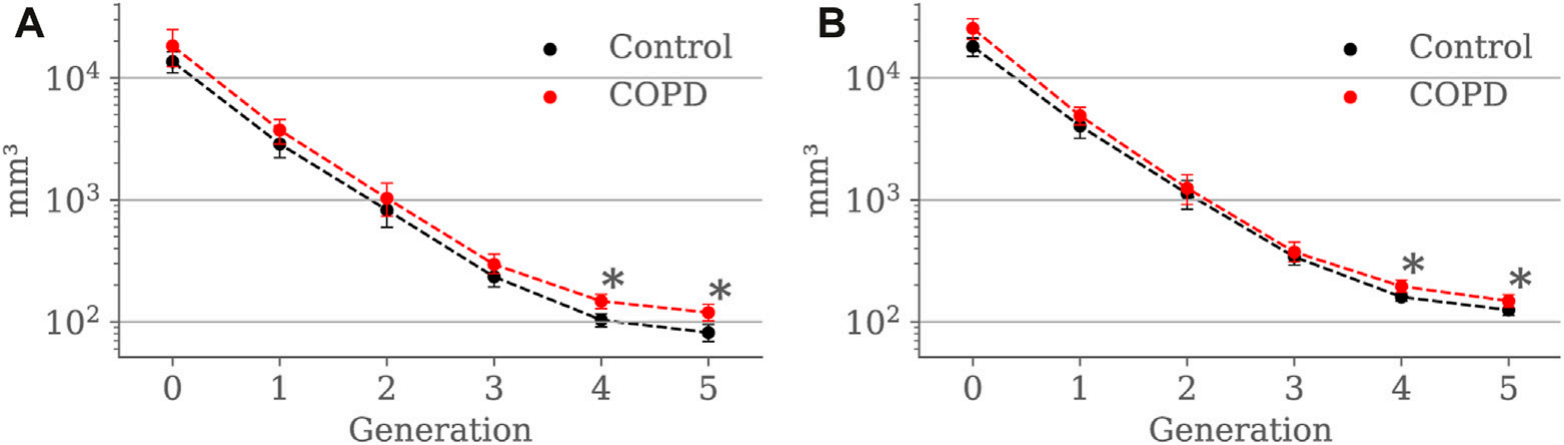
Airway luminal volume comparison between the Control and COPD groups from generation 0 to 5: **(A)** at EE, and **(B)** at EI. Dots represent mean values, and the error bars are confidence intervals. Nomenclature: *p < 0.05.

Figures 7A, B compare airway luminal volume for each generation at EE and EI, respectively. At EE, luminal volume was lower in the Control group for the 4*th* (103.6 ± 53.5 mm^3^ vs. 147.8 ± 90.6 *mm* ^3^, *p* < 0.001) and 5*th* (81.6 ± 53.9 mm^3^ vs. 119.3 ± 84.6 *mm* ^3^, *p* < 0.001) generation. At EI, airway luminal volume was also lower in the Control group for the 4*th* (159.9 ± 69.0 mm^3^ vs. 195.6 ± 125.9 *mm* ^3^, *p* = 0.012) and 5*th* (125.1 ± 97.8 mm^3^ vs. 148.3 ± 107.4 *mm* ^3^, *p* = 0.043) generation.

**FIGURE 8:**
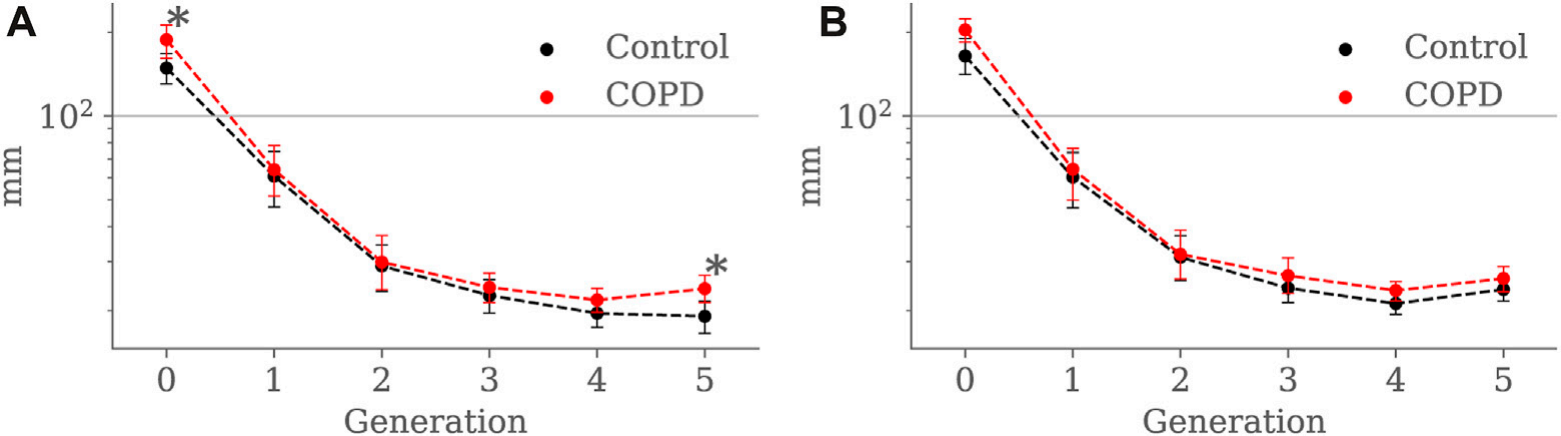
Airway segment length comparison between the Control and COPD groups from generation 0 to 5: **(A)** at EE, and **(B)** at EI. Dots represent mean values, and the error bars are confidence intervals. Nomenclature: *p < 0.05.

Figure 8 shows the airway segment length for each generation in both groups. At EE, airway length was higher in COPD patients for the trachea (148.6 ± 24.1 *mm* vs. 187.8 ± 31.2 *mm*, *p* = 0.041) and the 5*th* generation (19.0 ± 11.1 *mm* vs. 23.9 ± 13.0 *mm*, *p* = 0.013). At EI, no significant differences were found for any of the generations.

**FIGURE 9:**
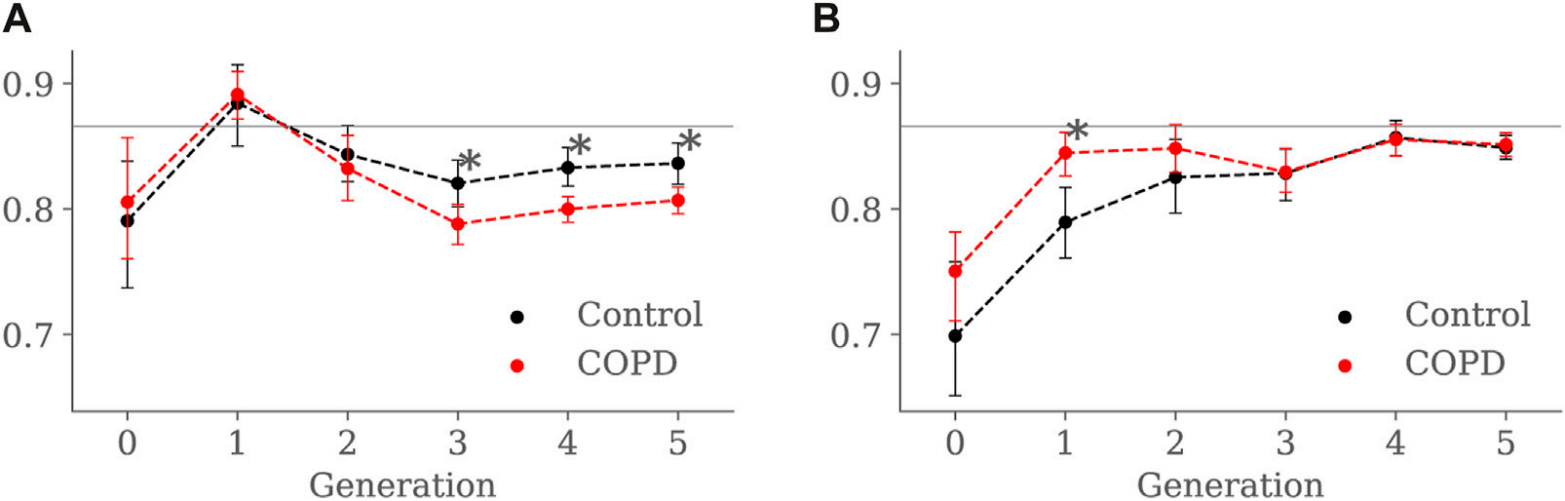
Airway luminal eccentricity comparison between the Control and COPD groups from generation 0 to 5: **(A)** at EE, and **(B)** at EI. Dots represent mean values, and the error bars are confidence intervals. Nomenclature: *p < 0.05.

Results for luminal eccentricity are reported in Figures 9A, B for the EE and EI states, respectively. We placed a horizontal line at 0.86, marking a relation of 1–2 times the radius, i.e., when the maximum radius is two times larger than the minimum radius. At EE, luminal eccentricity was higher in the Control group for the 3rd (0.82 ± 0.06 vs. 0.78 ± 0.05, *p* = 0.03), 4*th* (0.83 ± 0.06 vs. 0.79 ± 0.04, *p* = 0.004), and 5*th* (0.83 ± 0.06 vs. 0.81 ± 0.04, *p* = 0.005) generations. At EI, eccentricity was lower in the Control group but only for the 1st generation 1*st* (0.78 ± 0.05 vs. 0.84 ± 0.03, *p* = 0.007).

**FIGURE 10:**
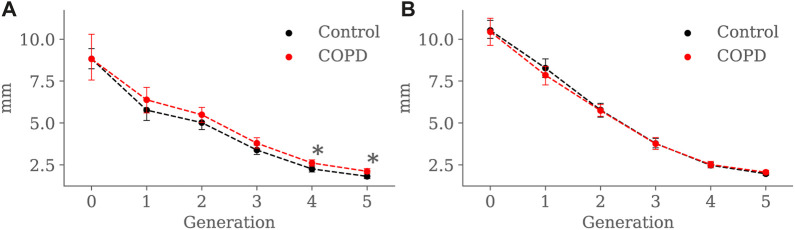
Airway minimum radius comparison between the Control and COPD groups from generation 0 to 5: **(A)** at EE, and **(B)** at EI. Dots represent mean values, and the error bars are confidence intervals. Nomenclature: *p < 0.05.

**FIGURE 11:**
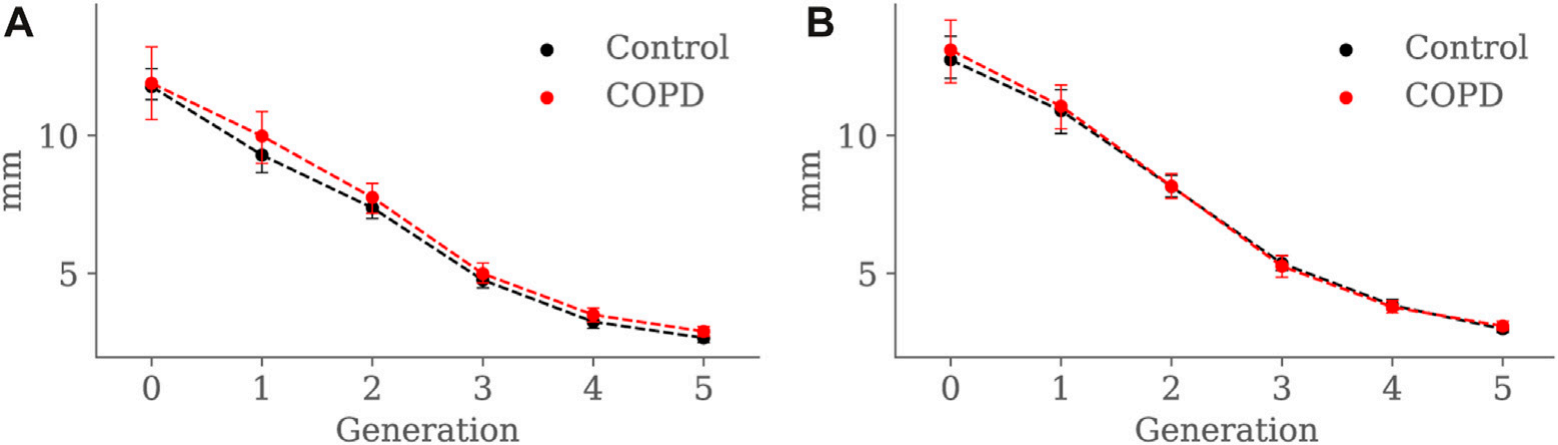
Airway average radius comparison between the Control and COPD groups from generation 0 to 5: **(A)** at EE, and **(B)** at EI. Dots represent mean values, and the error bars are confidence intervals. Nomenclature: *p < 0.05.

**FIGURE 12:**
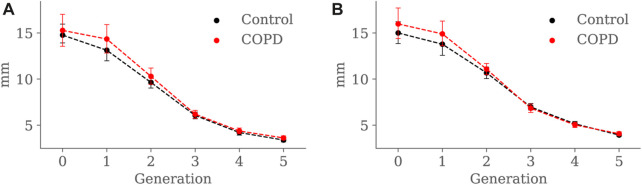
Airway maximum radius comparison between the Control and COPD groups from generation 0 to 5: **(A)** at EE, and **(B)** at EI. Dots represent mean values, and the error bars are confidence intervals. Nomenclature: * = *p* < 0.05.

Minimum, average, and maximum radii are shown in Figures 10–12, respectively. Only the minimum radii show significant differences between the Control and COPD groups for the 4*th* (2.2 ± 0.8 *mm* vs. 2.6 ± 0.8 *mm*, *p* = 0.03) and 5*th* (1.8 ± 0.6 *mm* vs. 2.1 ± 0.7 *mm*, *p* = 0.009) generations.

**FIGURE 13:**
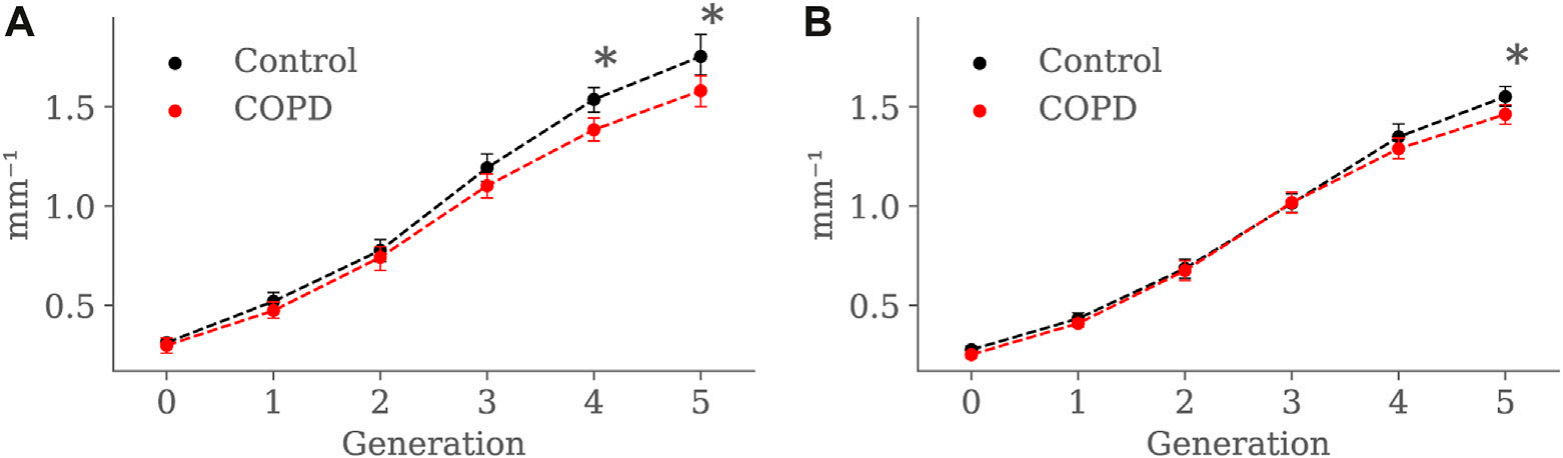
Airway SA:V ratio comparison between the Control and COPD groups from generation 0 to 5: **(A)** at EE, and **(B)** at EI. Dots represent mean values, and the error bars are confidence intervals. Nomenclature: * = *p* < 0.05.

Figures 13A, B present the SA:V ratio results at EE and EI, respectively. At EE, the SA: V ratio was significantly higher in the Control group in the 4*th* (1.5 ± 0.2 *mm* ^−1^ vs. 1.3 ± 0.2 *mm* ^−1^, *p* < 0.001) and 5*th* (1.7 ± 0.3 *mm* ^−1^ vs. 1.5 ± 0.3 *mm* ^−1^, *p* = 0.008) generations. At EI, the SA: V ratio was significantly higher in the Control group but only for the 5*th* generation (1.5 ± 0.3 *mm* ^−1^ vs. 1.4 ± 0.3 *mm* ^−1^, *p* = 0.012).

## 4 Discussion

This study examined the morphological differences in the airway tree among smoking individuals. Our database included smoking subjects divided into Control and COPD groups based on the severity classification criteria outlined in Labarca et al. (2017), Agustí et al. (2023). These groups displayed notable differences in pulmonary function, as reported in Table 1. The Control group, referred to as the pre-COPD group due to their smoking status (Ananth and Hurst, 2023), did not exhibit airflow limitations (FEV_1_/FVC ≥70%, post-bronchodilation) or symptoms (mMRC = 0). In contrast, the mild COPD group resulted in airflow obstruction (FEV_1_/FVC < 70%, post-bronchodilation) and clear symptoms (mMRC = 1 and 2). It is worth noting that both groups exhibited FEV_1_pred values greater than 80%. Consequently, the COPD group falls into the mild stage of disease severity (GOLD I) (GOLD, 2023).

We implemented the SIGA method, which utilizes the variational formulation of the Snakes problem to evolve NURBS surfaces and represent airway lumens, yielding improved performance compared to orthogonal projection techniques like Miyawaki et al. (2017), particularly in terms of the DICE coefficient. See Ortiz-Puerta et al. (2022) for a detailed comparison of these methods. The main findings of this study revealed significant differences in airway characteristics between the Control and COPD groups, particularly during different breathing states (EE and EI) and at specific generations of the airways. Lungs in the Control group displayed a higher TAC than the COPD group when comparing lungs in EI; see Figure 6A. Further reinforcement for this result is the marked reduction in EI GAC from the 5^*th*^ to 9^*th*^ generation in patients; see Figure 6B. These observations are consistent with previous studies, where comparisons between mild COPD patients and groups without airflow limitation or symptoms exhibited reductions of 17%–19% in the TAC of patients (McDonough et al., 2011; Kirby et al., 2018). TAC reduction could result from partial or complete airway blockage due to the lack of lung elastic tethering or increased airway stiffness resulting from ongoing inflammation in the lumen layers, which has been observed in COPD lungs (Eskandari et al., 2016). Thus, it is plausible that the airways are not missing but rather obliterated to the extent that they are no longer visible on CT scans (Díaz, 2018). Another interesting result is the decreasing number of GAC from generation 5 onwards in both groups. Using a dichotomous branching model of respiratory airways, which delivers an exponential law for GACs (2^*n*^ with *n* the generation number), one could predict a GAC of 32 at the 5*th* generation, which validates the GAC reached in the Control group for that generation (31.83). The limitations of CT resolution can explain the unexpected decreasing trend observed in both groups after the 5*th* generation. In effect, previous contributions have noted a similar tendency, attributing such reduction to the inability of CT images to resolve airways with lumen diameters smaller than the voxel resolution, typically around 1–2 *mm* (Diaz et al., 2015; Kirby et al., 2018).

Our study reveals a substantial increase in lumen volume for the COPD group, particularly for the 4*th* and 5*th* generations, both at EE and EI; see Figure 7. When comparing our findings to previous studies, we identify some discrepancies. Diaz et al. (2015) reported that never smokers experiencing lower FEV_1_ have reduced lumen volume, while Koyama et al. (2012b) reported a reduction of the luminal volume with the progression of the disease in terms of the GOLD classification. These results suggest a correlation between luminal volume reduction, expiratory airflow limitation, and disease severity. Therefore, the greater lumen volume observed in our patients with COPD seems counterintuitive because due to changes in intrinsic lung properties related to emphysema, loss of radial attachments to outer airway walls should produce lower distending forces acting on these peripheral airways, resulting in lower volume (Linhartová et al., 1971). However, thinner airway walls may predispose to airway closure, in which case the increased lumen volume at EE could represent trapped air above the point of collapse (Wilson et al., 1974) with consequent pathologic dilatation of the airways. Since the method used in our study does not allow for assessing airway wall thickness, we can only speculate at this point. Unfortunately, previous histological and imaging studies of proximal airway morphology have yielded conflicting results about airway wall thickness. Some studies on smokers have demonstrated mural thickening of the proximal airways, particularly in mild COPD (Tiddens et al., 1995; Grydeland et al., 2010). Conversely, more extensive imaging studies, including subjects with a broad spectrum of airflow obstruction, have found thinner-walled proximal airways in patients with COPD compared to Control subjects (Smith et al., 2014; Washko et al., 2014).

Our results indicate that airways in COPD patients tend to be longer than in Controls; see Figure 8. Further, in Table 1, we observe that lung volumes are more prominent in COPD patients than in Control subjects. These findings align with the tendency of lungs affected by COPD to be larger than normal lungs due to loss of lung elastic recoil, and the airways embedded in the lung tissue would elongate as the lung inflates, their length increasing by the cube root of lung volume (Tanabe et al., 2017). Even when some investigators have reported shorter airways in patients with COPD, those findings come from subjects with advanced emphysema (McDonough et al., 2011; Tanabe et al., 2017). In such circumstances, disruption of the acinar fiber networks reduces the tensile forces they exert on the proximal structures, mainly on the terminal and preterminal airways that will retract toward the larger, more central airways, decreasing length; at the same time, they will withdraw from the acinar structures, thus increasing the acinar space, the hallmark of emphysema (Mitzner, 2011; Weibel, 2013). Nevertheless, we believe that this may not be the case in our study, considering that our COPD group is at an early stage of the disease (GOLD I). Therefore, our patients’ airway lengthening could result from higher lung volumes associated with mild emphysema and passive hyperinflation, indicated by the increased LAA% and reduced DLCO; see Table 1.

For the COPD group at EE, less eccentric (i.e., more circular) airways along with higher length may be an expression of less frequent branching patterns at generations 3–5; see Figure 9A and Figure 8A, respectively. In the bronchial tree, the bifurcation point identifies the division of an airway, where the daughter branches separate. Frequent branching could make airway cross sections mostly non-circular (i.e., more eccentric), as shown by Eskandari et al. (2013). However, suppose the collapse of one of the two daughter branches. In that case, a bifurcation point cannot be identified, and the morphology of the remaining daughter branch is considered part of the main branch. The result is a spurious increase in the airway length, volume, and circularity (lower luminal eccentricity).Consequently, the greater volume, length, and circularity found in the airways of COPD patients at EE may represent less frequent branching patterns at generations 3 to 5; see Figure 7A and Figure 8A, respectively. Interestingly, Choi et al. (2015), Choi et al. (2017) reported a positive correlation between length and circularity with airway remodeling by chronic inflammation in COPD and asthma. Note that differences in the expiratory luminal eccentricity are due to the reduced minimum radius in Figure 10A for the same generations, considering that similar values are observed for the average and maximum radius; see Figure 11 and Figure 12, respectively.

When comparing groups at EI, Control subjects displayed less eccentric (more circular) proximal airways than the COPD subjects, see Figure 9B. Furthermore, the luminal eccentricity in the COPD group increased to the same values in the Control group from the 3^*rd*^ to 5^*th*^ generations. Consistent with our findings, Choi et al. (2017) reported similar tendencies for their Control and COPD group. In their study, Control subjects had more circular proximal airways, and the circularity was reduced while advancing to the distal generations. Further, they reported that the COPD group presented significantly lesser circularity for the proximal and distal airways but no significant differences for both groups at distal airways. The higher luminal eccentricity in the proximal airways of COPD patients may suggest that chronic bronchitis or inflammation-related airway remodeling affected large airways more than small ones (Choi et al., 2017).

At EE, the Control group exhibited higher SA: V than the COPD group for the 4*th* and 5*th* generations, as observed in Figure 13, but at EI, higher SA:V was only found in the 5*th* generation. As expected, there was a generation-to-generation increase in SA:V in both groups at EE and EI, which can be attributed to a greater loss in luminal volume than surface area as the airways divide towards the periphery, facilitating proper gas exchange (Bodduluri et al., 2021). Despite significant differences in the distal generations, characterizing airway remodeling in terms of narrowing or loss as described in Bodduluri et al. (2021) becomes challenging when studying SA:V by generation. When considering the airways as tubular structures, different combinations of length and radius can result in equivalent surface area and volume. Consequently, the same SA:V value can be obtained in both scenarios: when the airway shortens and thickens (possible loss) and when it lengthens and narrows (possible narrowing).

We perform a global study by adding the SA:V from all available airway segments for each subject and comparing the results for both groups. At EE, the global SA:V was 74.81 ± 42.91 *mm* ^−1^ and 77.33 ± 43.36 *mm* ^−1^ for the Control and COPD group, respectively. At EI, we observed significant differences between the Control and COPD groups, with values of 210.98 ± 39.49 *mm* ^−1^ and 129.52 ± 28.97 *mm* ^−1^ (*p* < 0.026), respectively. In accordance with our global results, Bodduluri et al. (Bodduluri et al., 2021) also reported significant differences for COPD GOLD stages 0 and I-II, which correspond to our Control and COPD groups, respectively. Notably, these global results could be associated with the TAC, as depicted in Figure 6A, where significant differences were exclusively observed at EI. In the same study, Bodduluri et al. found that individuals with predominant airway loss had lower TAC than those with predominant airway narrowing. The link between TAC and global SA:V can be explained by the global measure’s reliance on summing this value from each airway. Such a relationship is strengthened when accounting for a larger number of small airways at distal generations, where higher SA:V values are observed; see Figure 13.

The present study has certain limitations that should be addressed in future contributions. First, we considered a small number of patients for each of the groups analyzed. To address this, future research should include a larger cohort, comprising a non-smoking Control group, as well as groups representing various stages of disease progression and severity (GOLD I to IV), as proposed in previous studies by Kirby et al. (2018), Kirby et al. (2021). Furthermore, the Mann-Whitney test is prone to type II errors when used with a reduced sample size. Consequently, expanding the patient cohort can be a practical step to reduce this type of error. Second, our method relies on a semi-automated airway segmentation process using the ITK-SNAP software. Manual seeds are placed on visually identified airways to create the level-set-based binary mask. However, the seed placement may be affected by resolution limitations, posing challenges in accurate airway identification. To enhance precision, researchers should consider alternative approaches involving fully automated tools or commercial software for segmentation, such as the one mentioned in Diaz-Pinto et al. (2022). Third, our approach does not assess bronchial wall thickness to validate our findings. To address this limitation, future studies should incorporate previously used markers such as Wall Area (WA) and Lumen Area (LA) and perform comparative analyses. A feasible approach to assess airway thickness with our SIGA method is by adapting the “Full-width at half maximum” (Coxson, 2008) for airway thickness assessment using NURBS surfaces. This can be achieved by projecting normal vectors from the surface and measuring the image intensity. Moreover, the functional formulation of the SIGA method offers versatility, not only in controlling the surface evolution but also in adapting mechanical-related regularizers for studying bronchial wall mechanical deformation, as demonstrated in recent studies (Cox et al., 2022). Such a study could contribute to regional understanding of deformation in the ariways, as done in the past for the lung tissue Hurtado et al. (2017). Fourth, to avoid marker biases, it is crucial to consider the addition of normalization schemes in our research. This is particularly important as observed in asthma studies where conflicting results have been reported due to the lack of demographic normalization schemes (Choi et al., 2015). Finally, our study lacks a comparison of the morphology characterization with respect to pulmonary function tests and the quantification of emphysema. Future work should explore correlations between morphological changes, the airflow reduction in the groups under consideration, and the severity of emphysema.

## Data Availability

The original contributions presented in the study are included in the article/Supplementary Material, further inquiries can be directed to the corresponding author.
